# Supplementary material for: Performance and usability of cardiometabolic point of care devices in Nepal: A prospective, quantitative, accuracy study
Source: PLOS Glob Public Health. 2024 Oct 3;4(10):e0003760. doi: 10.1371/journal.pgph.0003760 (PMC11449279; doi:10.1371/journal.pgph.0003760)
Supplement: Attachment — Submitted filename: Response to Reviewers.docx [file pgph.0003760.s003.docx]

**Rebuttal letter**

PGPH-D-24-00096
Performance and usability of cardiometabolic point of care devices in Nepal: a prospective, quantitative, accuracy study
PLOS Global Public Health

3^rd^ April 2024

Dear Dr. Cowley,

We would like to thank the reviewer and editor for their feedback and seeing value in our work. We also appreciate your key insights and comments on this paper.

Kindly find below detailed responses to the comments sent by the reviewer and we do hope that our answers will address the remaining concerns.

We are of course willing to make further changes if necessary and we remain available for any additional questions.

Thank you again for this opportunity to share our work and to receive the feedback from your review committee.

We are looking forward to your response and hoping for a final positive outcome.

Yours sincerely,

*Marina Giachino*

Division of Tropical and Humanitarian Medicine

Geneva University Hospitals, Geneva, Switzerland

[Marina.Giachino@hcuge.ch](mailto:Marina.Giachino@hcuge.ch)

| **Journal’s requirements** | **Authors’ response** | **Information in the Revised Manuscript** |
| --- | --- | --- |
| 1. Please send a completed 'Competing Interests' statement, including any COIs declared by your co-authors. If you have no competing interests to declare, please state "The authors have declared that no competing interests exist". Otherwise please declare all competing interests beginning with the statement "I have read the journal's policy and the authors of this manuscript have the following competing interests:" | The information has been updated according to the journal’s policy, on p.17   - Marina Giachino, Zoltan Pataky, Olivia Heller, Bruno Lab, Jorge César Correia, Sanjib Kumar Sharma, Vijay Kumar Khanal, Sagar Poudel, Mamit Rai declared that no competing interests exist regarding the work. - I have read the journal’s policy and the following authors: Beatrice Vetter, Berra Erkosar have the following competing interests: they are both employees of FIND. As for the author: Sigiriya Aebischer Perone, she has the following competing interests: is an employee of ICRC, the International Committee of the Red Cross   Is the COIs stated at the end of the manuscript sufficient? | P.17 of Revised Manuscript:  "Competing interests statement  Marina Giachino, Zoltan Pataky, Olivia Heller, Bruno Lab, Jorge César Correia, Sanjib Kumar Sharma, Vijay Kumar Khanal, Sagar Poudel, Mamit Rai declared that no competing interests exist regarding the work.  Beatrice Vetter, Berra Erkosar have the following competing interests: they are both employees of FIND.  Sigiriya Aebischer Perone has the following competing interest: is an employee of ICRC, the International Committee of the Red Cross.” |
| 2. We have noticed that you have uploaded Supporting Information files, but you have not included a list of legends. Please add a full list of legends for your Supporting Information files after the references list. | p.19 As requested, a full list of legends for the Supporting Information files has been added after the references list. | P.19: “Supporting Information Files  1.S1 Checklist. STARD Checklist for accuracy studies  2.S2 Protocol. Clinical Study Protocol  3.S3 Dataset. Final Study Dataset” |
| 3. In the online submission form, you indicated that "The data will be made available by the corresponding author upon reasonable request". All PLOS journals now require all data underlying the findings described in their manuscript to be freely available to other researchers, either 1. In a public repository, 2. Within the manuscript itself, or 3. Uploaded as supplementary information. This policy applies to all data except where public deposition would breach compliance with the protocol approved by your research ethics board. If your data cannot be made publicly available for ethical or legal reasons (e.g., public availability would compromise patient privacy), please explain your reasons by return email and your exemption request will be escalated to the editor for approval. Your exemption request will be handled independently and will not hold up the peer review process, but will need to be resolved should your manuscript be accepted for publication. One of the Editorial team will then be in touch if there are any issues. | The raw dataset has been uploaded as supplementary information and is made publicly available. A legend excel sheet was added to the raw data to ensure overall understanding. | P.19: “Supporting Information Files  1.S1 Checklist. STARD Checklist for accuracy studies  2.S2 Protocol. Clinical Study Protocol  3.S3 Dataset. Final Study Dataset” |
| **Comments from Reviewer:** | **Authors’ response** | **Information in the Revised Manuscript** |
| **Reviewer #1: Interesting manuscript addressing an important global need. To improve scientific clarity, please address below comments** | Thank you for your review and positive comment. We have tried to address all the comments below in order to improve the manuscript. |  |
| 1. All figures are of a poor quality | We uploaded our figure files to the Preflight Analysis and Conversion Engine (PACE) digital diagnostic tool, <https://pacev2.apexcovantage.com/>. to ensure that figures meet PLOS requirements.  Is this sufficient or would you like us to re-draw the figures? | Updated figures are uploaded to the manuscript based on PACE digital diagnostic tool’s correction and conversion. |
| 2. Has the protocol been published? | The protocol information is now publicly available on ClinicalTrials.gov (NCT05257564) <https://clinicaltrials.gov/study/NCT05257564>  It has also been uploaded as supporting information file. | Information in the manuscript about the protocol availability:  p. 1: “**Trial registration:** NCT05257564 (ClinicalTrials.gov)”  p.8: “This study was conducted in accordance with the protocol (available under ClinicalTrials.gov)”  P.19: “Supporting Information Files  2. S2 File. Clinical Study Protocol” |
| 3. Has the authors considered the relevance of learning curve? | The relevance of learning curve has been considered throughout the System Usability Scale (SUS) in two aspects (a=response bias and b=learning curve needed for the system): a) users were asked to complete the SUS at the end of the study when any learning effect in system-use that may have affected their scoring would have taken place; b) question 7 of the SUS asks the user if they “would imagine that most people would learn to use this system very quickly”, which leads to a rating on the learning curve associated with the use of the system; question 10 of the SUS asks the user if they “needed to learn a lot of things before [they] could get going with the system”, which helps to judge the learning curve required on a system. | In addition to the results of the SUS in table 1, the text on p.13 highlights the SUS outcomes: “There was however a strong agreement that the system required a lot of training before use (average rating 4.2/5)".  We have added a sentence to the discussion text on p.15 to highlight the potential learning required “While users mostly felt that most people would learn to use both systems quickly, they judged the learning required to be substantial prior to first system use.” |
| 4. What are the essential characteristics for the point of care devices as presented in TPP. Author mentioned 41 characterstics in total, are there any key characteristics? Would be good to know how these two devices have been chosen. | They key characteristics for the selection were:  - suitable for use in primary health care in low resource settings  -operational at high temperature (≥35°C)  - minimal training requirements for the use of the devices  -self-contained cartridges or strips  For more information on the characteristics:  <https://bmccardiovascdisord.biomedcentral.com/articles/10.1186/s12872-021-02298-7>  For more information on how the devices were chosen:  <https://www.finddx.org/wp-content/uploads/2023/02/20200228_rep_landscape_poc_cardiometabolic_diseases_FV_EN.pdf>  These two documents are cited under the references. | We expanded the explanation of characteristics in focus for product selection on page 3-4 as follows:  “Using the TPP requirements to assess devices in the current landscape [2], two devices were identified that met several of these criteria *(and particularly TPP characteristic number 1-5, 10, 12, 23, 24 and 26, describing requirements of the intended use, target setting and operators, device design, environmental stability, training time, test menu, test cartridges and need for additional consumable [55])*:….” |
| 5. How was the quality control carried out? By whom, using what standard to ensure the device is operating safety and accurately? | The quality control of the POC testing was performed in each site for each parameter once a week by the same laboratory staff, according to the manufacturer’s guidelines and control solutions provided in order to control that the target values shared by the manufacturers are reached.  For reference laboratory testing, the B.P. Koirala Institute of Health Sciences used a Roche Cobas c311 analyser, according to the manufacturer’s instructions performing all local quality control/calibration standard operating procedures, by the reference laboratory staff. | p. 6: “Quality control testing for each parameter was performed by the study’s laboratory staff, once per week using the manufacturers’ control solutions and guidelines with the target values to be reached”.  p. 6: “the B.P. Koirala Institute of Health Sciences laboratory using a Roche Cobas c311 analyser [11], according to the manufacturer’s instructions and performing all local quality control/calibration standard operating procedures” |
| 6. Was there any study in terms of how methods and timing treating and analysing samples result in the discrepancies (biases) as observed in this study? | Studies have been conducted to estimate glucose degradation kinetics and work by Stapelton et al. has shown that glycolysis at room temperature is minimal in the first 30 min after blood collection, thus the bias introduced in our study is considered to be minimal. | We have added this as a further potential limitation to p15: “Degradation of glucose through glycolysis in the reference sample could also be a potential source of positive bias observed in the study, however any effect was likely minimal (around 0.1 mmol/l or 1.8 mg/dL), based on findings of a previous study on glucose degradation kinetics in venous blood samples kept at room temperature for 30 min.^[1]^”  Furthermore, we have added the type of anti-coagulant used in the plasma collection tube to p.6: “Plasma *in K3-EDTA collection tubes* was prepared from venous blood” |
| 7. What are the consequences of the bias? The author mentioned clinically significant, insignificant, but would be good to know the details. for instance, what would a high HbA1C reading lead to? | Measurement of HbA1c have important clinical implications for monitoring therapy, calculating the eAG, and diagnosing diabetes.  **General comments:**  Each 1% reduction in updated mean HbA1c was associated with reductions in risk of 21% for any end point related to diabetes (95% confidence interval 17% to 24%, P < 0.0001), 21% for deaths related to diabetes (15% to 27%, P < 0.0001), 14% for myocardial infarction (8% to 21%, P < 0.0001), and 37% for microvascular complications (33% to 41%, P < 0.0001).^[2]^ Therefore, measuring HbA1c has implication in the management and prevention of complication of diabetes. False low measurements can erroneously imply that diabetes is under control, while false high measurements can justify stricter control measures that increase the risk of hypoglycemia and its aftereffects. As a matter of fact, people with type 2 diabetes find it challenging to achieve and sustain levels of HbA1c that are close to normal, especially if their initial HbA1c concentration was high.^[3]^ Consequently, it is unlikely to cause harm to individuals on a communal level.  **Diagnostic implication:**  In a series of 12,785 male diabetic patients, *Khan et al.* have shown that the HbA1c cut-point of 6.5% was associated with 3.78% false-negative predictions, while majority of the false-negative patients had borderline FPG (7.0–8.0 mmol/L) and HbA1c (6.0%–6.5%), and therefore belonged to at-risk category on the basis of HbA1c alone criteria. These findings suggest that the status of individuals with HbA1c between 6.0% and 6.5% should be verified by combined FPG and HbA1c criteria^[4]^.  **Prognostication implication:**  A community-based population study on 11,092 nondiabetic patients found that elevated HbA1c level was strongly associated with the risk of cardiovascular disease and mortality.^[5]^ Even an increase of 1% in HbA1c concentration was associated with about 30% increase in all-cause mortality and 40% increase in cardiovascular or ischemic heart disease mortality, among individuals with diabetes. Whereas reducing the HbA1c level by 0.2% could lower the mortality by 10%.^[6]^ | P.14: “However, some parameters did show positive bias (>10% difference versus laboratory results) that exceeded RCPA/CLIA thresholds, such as HbA1c with JCAina and glucose and total cholesterol with TAS101. *Measuring HbA1c, for example, has clinical implication in the management and prevention of diabetes complication. False low measurements can erroneously imply that diabetes is under control, while false high measurements can justify stricter control measures that would increase the risk of hypoglycemia and its aftereffects.* As such, it is critical that the level of acceptable bias is established before making decisions about point-of-care device implementation and use.”  p.10: “For HbA1c, a moderate (10–20%) positive bias was observed, which exceeded the RCPA/CLIA recommended thresholds (± 6–8%) [17,18].” |
| 8. are the study population the intended patient groups? The inclusion, exclusion criteria are not clear. More details, such as the protocol, would help, laying out the essential PICO question the patients, the user, the comparator, the outcomes for the study. | The protocol is made publicly available under clinicatrials.gov. All of the information below is stated in the protocol.  The study population are members of the local community and facility attendees in the context of the KHDC-Nepal early detection program, under which they have access to the relevant healthcare related to any clinical findings from this diagnostic accuracy study.  **The inclusion criterias:**  Participants are eligible to be included in the study only if all of the following inclusion criteria apply:  - 20 years or older (in line with KHDC eligibility age)  - Attending the Dhulabari PHC facility or Kakarvitta health post in the context of the KHDC program  - Haemoglobin levels ≥8 g/dL  - Able and willing to provide informed consent  **The exclusion criterias:**  Participants are excluded from the study if any of the following exclusion criteria apply:  - Inability to provide sufficient capillary or venous whole blood sample for all tests  - Haemoglobin levels < 8g/dL  - Anyone attending the Dhulabari PHC facility or Kakarvitta health post for other reasons than the KHDC program | In addition to the stated inclusion and exclusion criteria in p.5 we have provided more information related to the KHDC patient group and therefore about our study population.  P.5: “The study was embedded in the KHDC-Nepal program, which has the objective to improve the health of the Nepalese population in Eastern Nepal through early detection and management of chronic noncommunicable diseases. The program runs community awareness sessions and door-to-door visits to encourage members of the community to attend a screening visit at selected primary health facilities, health posts or at a pre-designated location. During this visit, people are screened for hypertension, diabetes, chronic kidney and cardiovascular disease, including (but not limited to) testing for glucose, lipids, HbA1c and creatinine, therefore the participants of this study are the intended use population for the point of care devices.” |

While revising your submission, please upload your figure files to the Preflight Analysis and Conversion Engine (PACE) digital diagnostic tool, <https://pacev2.apexcovantage.com/>. PACE helps ensure that figures meet PLOS requirements. To use PACE, you must first register as a user. Registration is free. Then, login and navigate to the UPLOAD tab, where you will find detailed instructions on how to use the tool. If you encounter any issues or have any questions when using PACE, please email PLOS at [figures@plos.org](mailto:figures@plos.org). Please note that Supporting Information files do not need this step.

- Authors: The figures were uploaded to the Preflight Analysis and Conversion Engine (PACE) digital diagnostic tool and all of the figures met PLOS requirements.

1. *Stapleton M, Daly N, O’Kelly R, Turner MJ. Time and temperature affect glycolysis in blood samples regardless of fluoride-based preservatives: a potential underestimation of diabetes. Annals of Clinical Biochemistry. 2017;54(6):671-676. doi:10.1177/0004563216682978*
2. *Irene M Stratton, Amanda I Adler, H Andrew W Neil, David R Matthews, Susan E Manley, Carole A Cull, David Hadden, Robert C Turner, Rury R Holman on behalf of the UK Prospective Diabetes Study Group. Association of glycaemia with macrovascular and microvascular complications of type 2 diabetes (UKPDS 35): prospective observational study. BMJ 2000;321:405–12*
3. *UKPDS Group. UK prospective diabetes study 24: relative efficacy of sulfonylurea, insulin and metformin therapy in newly diagnosed non-insulin dependent diabetes with primary diet failure followed for six years. Ann Intern Med 1998;128:165-75.*
4. *Khan HA, Ola MS, Alhomida AS, Sobki SH, Khan SA. Evaluation of HbA1c criteria for diagnosis of diabetes mellitus: a retrospective study of 12785 type 2 Saudi male patients. Endocr Res. 2014;*39*:62–6.*
5. *Martin-Timon I, Sevillano-Collantes C, Segura-Galindo A, del Cañizo-Gómez FJ. Type 2 diabetes and cardiovascular disease: have all risk factors the same strength?* World J Diabetes. *2014;5:444–70*
6. *Khaw KT, Wareham N, Luben R, et al. Glycated haemoglobin, diabetes, and mortality in men in Norfolk cohort of European Prospective Investigation of Cancer and Nutrition (EPIC-Norfolk) BMJ. 2001;322:15–8*
