# Supplementary material for: Performance and usability of cardiometabolic point of care devices in Nepal: A prospective, quantitative, accuracy study
Source: PLOS Glob Public Health. 2024 Oct 3;4(10):e0003760. doi: 10.1371/journal.pgph.0003760 (PMC11449279; doi:10.1371/journal.pgph.0003760)
Supplement: Attachment — Submitted filename: Response to Reviewers.docx [file pgph.0003760.s004.docx]

**Rebuttal letter**

PGPH-D-24-00096
Performance and usability of cardiometabolic point of care devices in Nepal: a prospective, quantitative, accuracy study
PLOS Global Public Health

16^th^ of July 2024

Dear Dr. Kyobutungi and Prof. Pai,

We would like to thank the reviewer for the feedback and seeing value in our work. We also appreciate your key insights and comments on this paper.

Kindly find below detailed responses to the comments sent by the reviewer and we do hope that our answers will address the remaining concerns.

We are of course willing to make further changes if necessary and we remain available for any additional questions.

Thank you again for this opportunity to share our work and to receive the feedback from your review committee.

We are looking forward to your response and hoping for a final positive outcome.

Yours sincerely,

*Marina Giachino*

Division of Tropical and Humanitarian Medicine

Geneva University Hospitals, Geneva, Switzerland

[Marina.Giachino@hcuge.ch](mailto:Marina.Giachino@hcuge.ch)

| **Journal’s requirements** | **Authors’ response** | **Information in the Revised Manuscript** |
| --- | --- | --- |
| 1. Please review your reference list to ensure that it is complete and correct. If you have cited papers that have been retracted, please include the rationale for doing so in the manuscript text or remove these references and replace them with relevant current references. Any changes to the reference list should be mentioned in the rebuttal letter that accompanies your revised manuscript. If you need to cite a retracted article, indicate the article’s retracted status in the References list and also include a citation and full reference for the retraction notice. | The reference list has been reviewed and we confirm the list is complete and correct.  We have verified, and all articles are still accessible and relevant, none were retracted. During the last submission, one additional reference was added after one of the first reviewer’s comment. And based on the second reviewer, additional references were inserted to support the additional information mentioned in the manuscript.  We have detailed here, the new references added. | P.18-22  5. Albitres-Flores L, Perez-Leon S, Bernabe-Ortiz A, Tenorio-Mucha J, Cardenas MK, Vetter B, et al. Co-creation process of an intervention to implement a multiparameter point-of-care testing device in a primary healthcare setting for non-communicable diseases in Peru. BMC Health Services Research. 2024;24(1):401. doi: 10.1186/s12913-024-10809-3.  6. Tenorio-Mucha J, Busta-Flores P, Lazo-Porras M, Vetter B, Safary E, Moran AE, et al. Facilitators and barriers of the implementation of point-of-care devices for cardiometabolic diseases: a scoping review. BMC Health Serv Res. 2023;23(1):412. Epub 20230428. doi: 10.1186/s12913-023-09419-2. PubMed PMID: 37118750; PubMed Central PMCID: PMCPMC10144879.  13. Sacks DB, Arnold M, Bakris GL, Bruns DE, Horvath AR, Kirkman MS, et al. Executive Summary: Guidelines and Recommendations for Laboratory Analysis in the Diagnosis and Management of Diabetes Mellitus. Clinical Chemistry. 2011;57(6):793-8. doi: 10.1373/clinchem.2011.163634.  24. Khan AI, Pratumvinit B, Jacobs E, Kost GJ, Kary H, Balla J, et al. Point-of-care testing performed by healthcare professionals outside the hospital setting: consensus based recommendations from the IFCC Committee on Point-of-Care Testing (IFCC C-POCT). Clinical Chemistry and Laboratory Medicine (CCLM). 2023;61(9):1572-9. doi: doi:10.1515/cclm-2023-0502.  25. Hirst JA, McLellan JH, Price CP, English E, Feakins BG, Stevens RJ, et al. Performance of point-of-care HbA1c test devices: implications for use in clinical practice - a systematic review and meta-analysis. Clin Chem Lab Med. 2017;55(2):167-80. doi: 10.1515/cclm-2016-0303. PubMed PMID: 27658148.  26. Nataatmadja M, Fung AWS, Jacobson B, Ferera J, Bernstein E, Komenda P, et al. Performance of StatSensor Point-of-Care Device for Measuring Creatinine in Patients With Chronic Kidney Disease and Postkidney Transplantation. Can J Kidney Health Dis. 2020;7:2054358120970716. Epub 20201112. doi: 10.1177/2054358120970716. PubMed PMID: 33240520; PubMed Central PMCID: PMCPMC7672720.  27. Park PH, Chege P, Hagedorn IC, Kwena A, Bloomfield GS, Pastakia SD. Assessing the accuracy of a point-of-care analyzer for hyperlipidaemia in western Kenya. Trop Med Int Health. 2016;21(3):437-44. Epub 20160107. doi: 10.1111/tmi.12653. PubMed PMID: 26663749.  28. FIND. Landscape of HbA1c Point-of-care testing devices. Available from https://www.finddx.org/wp-content/uploads/2022/12/20211101_lds_hba1c_poc_FV_EN.pdf (accessed 4 July 2024). 2021.  30. Carstensen B. Chapter 9 Repeatability, Reproducibility and Coefficient of Variation. Comparing Clinical Measurement Methods: A Practical Guide: John Wiley & Sons; 2010.  31. Clinical and laboratory standards institute (CLSI). EP05-A3 Evaluation of Precision of Quantitative Measurement Procedures, 3rd Edition (2014) |
| (22^nd^ of July)  Some material included in your submission may be copyrighted. According to PLOS’s copyright policy, authors who use figures or other material (e.g., graphics, clipart, maps) from another author or copyright holder must demonstrate or obtain permission to publish this material under the Creative Commons Attribution 4.0 International (CC BY 4.0) License used by PLOS journals. Please closely review the details of PLOS’s copyright requirements here: PLOS Licenses and Copyright. If you need to request permissions from a copyright holder, you may use PLOS's Copyright Content Permission form.  Please respond directly to this email or email the journal office and provide any known details concerning your material's license terms and permissions required for reuse, even if you have not yet obtained copyright permissions or are unsure of your material's copyright compatibility.  Potential Copyright Issues: "S2_Protocol. Clinical Study Protocol.pdf" contains branding/a logo. We are not permitted to publish this under our CC-BY 4.0 license, even with permission. We ask that you please remove or replace it. | We had added the Protocol as supplementary material upon request of the reviewer. However, since the protocol is already publicly available Online on [ClinicalTrials.gov](https://ClinicalTrials.gov) (<https://clinicaltrials.gov/study/NCT05257564?cond=NCT05257564>) and in the manuscript we do mention where to find the protocol (P.2/P.8), we have removed the document from the supplementary materials.  If you would prefer that we just retrieve the branding/logo but keep the document as supplementary materials, we will do so. | P. 22  Protocol was removed from the Supporting information Files:  1. S1 Checklist. STARD Checklist for accuracy studies  2. S2 Dataset. Final Study Dataset |
| (22^nd^ of July)  We note your Financial Disclosure statement:"This study was funded by FIND ([www.finddx.org](https://www.finddx.org)), through a grant from the International Committee of the Red Cross and the German Federal Ministry of Education and Research (NCT05257564; grant numbers: KFW-TBBU02; ICR-NCDS01). The study or authors were not funded by any commercial companies, no authors received a salary or other funding from commercial companies, and the funders had no role in the study design, data collection and analysis, decision to publish, or the preparation of the manuscript."  Before we proceed, please clarify which author(s) are the recipient(s) of the funding awards listed above.  Once we receive this information we will update your Financial Disclosure statement on your behalf. | For the Financial Disclosure: the author who was the recipient of the funding was Dr. Beatrice Vetter (from FIND) | No changes were done to our submission regarding this point since you stated “Once we receive this information we will update your Financial Disclosure statement on your behalf.” |
| **Comments from Reviewer:** | **Authors’ response** | **Information in the Revised Manuscript** |
| **Reviewer #2:** With interest I read the paper by Giachino et al. describing results of a cross-sectional accuracy study of prospectively recruited participants examining the performance of two different point-of-care devices assessing glucose, total cholesterol and glycated hemoglobin. While the concept is not novel, this manuscript provides information on two "new" devices in a distinct patient population from Nepal. Overall, the manuscript is well written, the methodology seems robust, and the statistical analyses are appropriately done and presented. Results are comprehensive and detailed.  I have some minor additional comments to the authors to improve their manuscript. | Thank you for your review and positive comment. We have tried to address all the comments below in order to improve the manuscript. | N/A. |
| 1. Please detail the time elapsed between venous blood collection and laboratory assessment of the markers of interest. This is especially important for glucose, as the longer the wait is, the longer chances of glucose decay is.  Also, were all analyses performed in one core lab? several sites? If so what was the coefficient of variance of the machines used for analyses in each site? | Blood samples were centrifuged for plasma separation within 30 minutes of venous blood collection in the clinic, to minimize glycolysis. This procedure is recommended by the American Diabetes Association in the *Guidelines and Recommendations for Laboratory Analysis in the Diagnosis and Management of Diabetes Mellitus.^[1]^*  The time between venous blood collection and laboratory assessment was kept to a minimum. After collecting the samples, they were stored at 2-8°C and a daily transport to the reference laboratory was organized. The samples were then tested the same day or the next morning. Throughout the transport and until tested, the samples were stored at 2-8°C.  All reference testing was performed in the same laboratory (we assume that the reviewer means the reference laboratory when referring to “core lab”), the B.P. Koirala Institute of Health Sciences laboratory. While the reviewer is likely asking for coefficient of variation (CV) to understand potential variation of reference results due to analysis in different laboratories (which was not the case in our study, as reference tests were only performed in a single laboratory on the same analyzer), we would still like to provide the reviewer with information on the CV (repeatability) for the reference tests run on the Roche Cobas c311 analyser and the respective parameters, for information (based on the Instructions for Use per parameter): CV for glucose, cholesterol and HbA1c is <2% and CV for creatinine is ≤4%.**^[2]^** | P.6  We have added the reference to the guidelines to the manuscript, along with the rational. “*Plasma in K3-EDTA collection tubes* was prepared from venous blood by centrifugation within 30 minutes of sample collection *to minimize glycolysis* and serum was prepared by centrifugation after the blood samples had been left to clot for up to 60 minutes.”***^1^***  We have provided more information in the manuscript related to the time elapsed between venous blood collection and laboratory assessment as well as the number of reference laboratory machine:  “All samples were then stored at 2–8^o^C until *transferred the same day to* the B.P. Koirala Institute of Health Sciences laboratory *and tested the same day or the next morning, using one* Roche Cobas c311 analyser [14], according to the manufacturer’s instructions and performing all local quality control/calibration standard operating procedures. *Before processing the samples at the reference laboratory, they were stored in cooling boxes with temperature monitoring throughout the transport and then stored at 2-8°C.”* |
| 2. To support agreement for continuous measurements between different devices/methods, please add an analysis for interclass correlation. | As all reference testing was performed on the same device, this assessment is not required. |  |
| 3. Were repeat measurements for intraclass correlation performed? Reliability is another important aspect not highlighted in the current manuscript. | Repeat measurements for intraclass correlation was not performed for the reference tests, based on the above-mentioned low CV for repeatability (see reviewer question 1). Appreciating that this is an important aspect to consider, we have added a sentence to the discussion section of the manuscript. | P.15  “The coefficient of variation (CV) for the reference tests are another source of variability in this study. However, based on the instructions for use by the manufacturer, the Roche cobas tests for glucose, cholesterol and HbA1c used in this study had a CV (repeatability) of <2% and ≤4% for creatinine, suggesting that the effect was minimal with a CV <5% often considered excellent.” **^[3],^^[4]^** |
| 4. Please discuss and compare the results to other existing devices as this field is saturated in widely available and relatively cheap devices. | Indeed, there are many cardiometabolic point-of-care devices available (as shown in a landscape FIND published in 2020)**^[5]^,** however, results of independent evaluations from other devices for all parameters tested in this study are not widely available. Published, manufacturer-independent accuracy studies for individual parameters suggest that performance can be highly variable in the field, deviating from accuracy claimed by the manufacturer. We have added a further point to the discussion section of the manuscript, following an existing paragraph comparing HbA1c performance of the investigational devices to other POC HbA1c devices. | P.14-15  “The International Federation of Clinical Chemistry recommends that “Device performance claims from the package insert should correlate with those obtained from verification studies that are performed by the [point-of-care testing] POCT personnel”**^[6]^.** This is critically important, considering variable field performance of point-of-care tests **^[7],^^[8],^^[9],^** the lack of manufacturer-independent evaluations and the high number of on-market cardiometabolic POC devices. »**^[10]^** |
| 5. Discussion should also focus on the gap which these devices are meant to bridge. The ability to perform point-of-care measurements of glucose and cholesterol is not novel. Are the devices in focus cheaper than alternatives? Are they easier to use? | The value of a multiparameter device has now been highlighted in the introduction by the addition of a sentence on P3.  Furthermore, these particular devices were selected as they meet several critical characteristics identified in the target product profile for cardiometabolic devices (which many others devices fall short of, based on the FIND landscape analysis):  - particularly high operating temperatures (≥35°C)  - ease of use (few-step handling process)  - ability to measure all four basic cardiometabolic tests on a single device  - ability to measure these tests in parallel (rather than sequential).  For more information on the characteristics:  <https://bmccardiovascdisord.biomedcentral.com/articles/10.1186/s12872-021-02298-7>  For more information on how the devices landscape:  <https://www.finddx.org/wp-content/uploads/2023/02/20200228_rep_landscape_poc_cardiometabolic_diseases_FV_EN.pdf>  These two documents are cited under the references. | P.3  “Measuring a combination of parameters on a single point-of-care device has the potential to facilitate a more comprehensive view of a patient’s condition by reducing the risk to forgo necessary tests due to the complexity of managing multiple point-of-care devices and supplies.”  The following sections in the manuscript already address these functional characteristics:  P.3-4  Besides measuring the key cardiometabolic parameters, devices should be suitable for use in primary health care in low resource settings, capable of operating at environmental temperatures of ≥35°C, be suitable for use by minimally trained healthcare workers, and involve the use of self-contained cartridges or strips without the need for additional reagents [7]. Using the TPP requirements to assess devices in the current landscape [2], two devices were identified that met several of these criteria *(and particularly TPP characteristic number 1-5, 10, 12, 23, 24 and 26, describing requirements of the intended use, target setting and operators, device design, environmental stability, training time, test menu, test cartridges and need for additional consumable [**7**]):* the JanaCare Aina Blood Monitoring System (JCAina) [8, 9] and the Tascom SimplexTAS 101 device (TAS101) [10, 11].  P.4  “However, as with many point-of-care devices, their accuracy has not been rigorously evaluated in their intended use setting and when operated by the intended users. Therefore, their accuracy in primary healthcare settings and routine use is unknown.” |

- Authors: The figures were uploaded to the Preflight Analysis and Conversion Engine (PACE) digital diagnostic tool and all of the figures met PLOS requirements.

1. David B Sacks, Mark Arnold, George L Bakris, David E Bruns, Andrea Rita Horvath, M Sue Kirkman, Ake Lernmark, Boyd E Metzger, David M Nathan, Executive Summary: Guidelines and Recommendations for Laboratory Analysis in the Diagnosis and Management of Diabetes Mellitus, Clinical Chemistry, Volume 57, Issue 6, 1 June 2011, Pages 793–798, <https://doi.org/10.1373/clinchem.2011.163634>
2. Instruction for Use for “GLUC2”, “CHOL2”, “A1C-3”, “CREJ2” Roche Cobas
3. CLSI EP05-A3 Evaluation of Precision of Quantitative Measurement Procedures, 3rd Edition (2014).
4. Carstensen B. Chapter 9 Repeatability, Reproducibility and Coefficient of Variation. Comparing Clinical Measurement Methods: A Practical Guide: John Wiley & Sons; 2010.
5. FIND. Landscape of point-of-care devices for testing of cardiometabolic diseases. Available from https://www.finddx.org/wp-content/uploads/2023/02/20200228_rep_landscape_poc_cardiometabolic_diseases_FV_EN.pdf (accessed 26 March 2023). 2020.
6. Khan AI, Pratumvinit B, Jacobs E, Kost GJ, Kary H, Balla J, et al. Point-of-care testing performed by healthcare professionals outside the hospital setting: consensus based recommendations from the IFCC Committee on Point-of-Care Testing (IFCC C-POCT). Clinical Chemistry and Laboratory Medicine (CCLM). 2023;61(9):1572-9. doi: doi:10.1515/cclm-2023-0502.
7. Hirst JA, McLellan JH, Price CP, English E, Feakins BG, Stevens RJ, et al. Performance of point-of-care HbA1c test devices: implications for use in clinical practice - a systematic review and meta-analysis. Clin Chem Lab Med. 2017;55(2):167-80. doi: 10.1515/cclm-2016-0303. PubMed PMID: 27658148.
8. Nataatmadja M, Fung AWS, Jacobson B, Ferera J, Bernstein E, Komenda P, et al. Performance of StatSensor Point-of-Care Device for Measuring Creatinine in Patients With Chronic Kidney Disease and Postkidney Transplantation. Can J Kidney Health Dis. 2020;7:2054358120970716. Epub 20201112. doi: 10.1177/2054358120970716. PubMed PMID: 33240520; PubMed Central PMCID: PMCPMC7672720.
9. Park PH, Chege P, Hagedorn IC, Kwena A, Bloomfield GS, Pastakia SD. Assessing the accuracy of a point-of-care analyzer for hyperlipidaemia in western Kenya. Trop Med Int Health. 2016;21(3):437-44. Epub 20160107. doi: 10.1111/tmi.12653. PubMed PMID: 26663749.
10. FIND. Landscape of HbA1c Point-of-care testing devices. Available from <https://www.finddx.org/wp-content/uploads/2022/12/20211101_lds_hba1c_poc_FV_EN.pdf> (accessed 4 July 2024). 2021.
